# DNA methylation and gene expression changes in mouse pre- and post-implantation embryos generated by intracytoplasmic sperm injection with artificial oocyte activation

**DOI:** 10.1186/s12958-021-00845-7

**Published:** 2021-11-04

**Authors:** Mingru Yin, Weina Yu, Wenzhi Li, Qianqian Zhu, Hui Long, Pengcheng Kong, Qifeng Lyu

**Affiliations:** 1 Department of Assisted Reproduction, Shanghai Ninth People’s Hospital, Shanghai Jiao Tong University School of Medicine, 200011 Shanghai, China; 2 Department of Assisted Reproduction, First Maternity and Infant Hospital, Tongji University School of Medicine, 201204 Shanghai, China

**Keywords:** Artificial oocyte activation, Mouse blastocysts, Gene expression, Imprinted gene, DNA methylation

## Abstract

**Background:**

The application of artificial oocyte activation (AOA) after intracytoplasmic sperm injection (ICSI) is successful in mitigating fertilization failure problems in assisted reproductive technology (ART). Nevertheless, there is no relevant study to investigate whether AOA procedures increase developmental risk by disturbing subsequent gene expression at different embryonic development stages.

**Methods:**

We used a mouse model to explore the influence of AOA treatment on pre- and post-implantation events. Firstly, the developmental potential of embryos with or without AOA treatment were assessed by the rates of fertilization and blastocyst formation. Secondly, transcriptome high-throughput sequencing was performed among the three groups (ICSI, ICSI-AOA and dICSI-AOA groups). The hierarchical clustering and Principal Component Analysis (PCA) analysis were used. Subsequently, *Igf2r/Airn* methylation analysis were detected using methylation-specific PCR sequencing following bisulfite treatment. Finally, birth rate and birth weight were examined following mouse embryo transfer.

**Results:**

The rates of fertilization and blastocyst formation were significantly lower in oocyte activation-deficient sperm injection group (dICSI group) when compared with the ICSI group (30.8 % vs. 84.4 %, 10.0 % vs. 41.5 %). There were 133 differentially expressed genes (DEGs) between the ICSI-AOA group and ICSI group, and 266 DEGs between the dICSI-AOA group and ICSI group. In addition, the imprinted gene, *Igf2r* is up regulated in AOA treatment group compared to control group. The *Igf2r/Airn* imprinted expression model demonstrates that AOA treatment stimulates maternal allele-specific mehtylation spreads at differentially methylated region 2, followed by the initiation of paternal imprinted *Airn* long non-coding (lnc) RNA, resulting in the up regulated expression of *Igf2r*. Furthermore, the birth weight of newborn mice originating from AOA group was significantly lower compared to that of ICSI group. The pups born following AOA treatment did not show any other abnormalities during early development. All offspring mated successfully with fertile controls.

**Conclusions:**

AOA treatment affects imprinted gene *Igf2r* expression and mehtylation states in mouse pre- and post-implantation embryo, which is regulated by the imprinted *Airn*. Nevertheless, no significant differences were found in post-natal growth of the pups in the present study. It is hoped that this study could provide valuable insights of AOA technology in assisted reproduction biology.

**Supplementary Information:**

The online version contains supplementary material available at 10.1186/s12958-021-00845-7.

## Introduction

Assisted reproductive technology (ART) provide a useful alternative for the infertility cases and five million children were born to date using these methods. The application of intracytoplasmic sperm injection (ICSI) has solved most male infertility problems in assisted reproductive medicine [1]. Whereas, 1–3 % of ICSI fertilization failure still occurs in ICSI cycles [2]. Oocyte activation deficiency (AOD) is one of the factors associated with ICSI failure. Oocyte activation is an imperative stage in the initiation of embryo development during the fertilization, involving mainly calcium (Ca^2+^) rise. The calcium oscillations are stimulated by the entrance of sperm, and then phosphatidylinositol 4,5-bisphosphate (PIP2) is hydrolyzed to inositol 1,4,5-trisphosphate (IP3) and diacylglycerol (DAG) by some sperm soluble factors. Whereafter, further Ca^2+^ oscillations are induced following IP3 binds to specific receptors. A series of molecular events such as exocytosis of cortical granules, recruitment of maternal mRNA, pronuclear formation, and polyspermy prevention occurred after oocyte activation [3–5]. Phospholipase C zeta (PLCζ) existed in sperm, which is considered to be the physiological factor for oocyte activation [6]. Apart from these natural activators, some stimulants, such as ionomycin, calcimycin, and strontium chloride have been applied to mitigate ICSI fertilization failure. These methods have been known as artificial oocyte activation (AOA) and are considered to be a useful method for fertilization failure problems.

With the increasingly wide application of AOA, some concerns remain regarding the safety of this technology. The period that oocyte activation is a critical window of gene imprinting removal, establishment and maintenance. So it is critically important to investigate whether AOA procedure increase developmental risks during the embryonic or neonatal development stages. Although some clinical data analyses have demonstrated that birth characteristics and congenital malformations following AOA are within the expected range [7–9], it is critically important to evaluate in details the impact of AOA in embryo early development from gene expression and epigenetic point of view. Therefore, it is essential to  animal models for further study. Mouse oocytes can be used to study oocyte activation in humans [10]. Therefore, a mouse model with oocyte AOD sperm would be useful for studying AOA. Some sperm-derived factors, such as PLCζ [11], are considered as the sperm-associated oocyte-activating factors (SOAFs). Some studies [12, 13] have proved that release of SOAFs from disrupted membranes had been shown to suppress oocyte activation. In our previous study, we established a simple and effective method for generating oocyte AOD mouse spermatozoa [14]. And with the release of SOAFs from disrupted membranes, spermatozoa with different oocyte activation-deficient were established.

Precise genomic imprinting plays an important role in regulating the growth and development of embryos, and also in the early postnatal phase. Manipulation or non-physiological culture environments may affect the gene expression or epigenetic modification of imprinted genes during early embryo development stages or later fetal development. DNA methylation, an epigenetic imprinting modification, acts in concert to negatively control gene expression. DNA methylation at differentially methylated regions (DMRs) or imprinting control regions (ICRs) is required for imprinted expression at many loci. But until now, it is unknown whether AOA treatment would produce some effects on gene expression and methylation in embryo development stages. Therefore, we aim to explore these in this study.

In the present study, transcriptome high-throughput sequencing was performed to provide a profile of the differences in the global patterns of gene expression between ICSI-AOA treatment and ICSI generated mouse blastocysts. In addition, we focused on the different expression of imprinted genes, of which the *Igf2r* was up-regulated in AOA treatment groups compared to ICSI group. It is known that the imprinted *Airn* long non-coding (lnc) RNA is paternally expressed and silences *Igf2r* genes in cis. Therefore, the study aims to explore the effect of AOA on the dynamic regulation of *Igf2r/Airn* in the pre- and post-implantation embryo.

## Materials and methods

### Animals

C57BL/6 mice were provided by the SLAC Laboratory Animal Co. Ltd (Shanghai, PRC) and were used to prepare oocytes and sperms. All animals were maintained under a 12:12 h lighting schedule rooms (lights on at 07:00-19:00). All animals were maintained and treated in accordance with the National Institutes of Health Guide for the Care and Use of Laboratory Animals and were approved by Animal Care and Use Committee of Shanghai Jiao Tong University School of Medicine.

### Mouse oocyte collection and activation-deficient spermatozoa handling

Mouse oocyte collection process was shown in our previous study [14, 15]. Female C57BL/6 mice (4–6 weeks old) were used for superovulation. Finally, the denuded oocytes were cultured in drops of KSOM (MR-107, Millipore, Germany) under mineral oil at 37 ℃ and 5 % CO_2_ in the incubator. An efficient method for producing activation-deficient mouse spermatozoa was identified in previous study [14]. Briefly, Epididymal spermatozoa were obtained from male C57BL/6 mice at 8–12 weeks. A dense sperm mass was incubated at 37℃ and 5 % CO_2_ for 30 min to gather motile sperm. A half of the top 1 ml sperm suspension was carefully aspirated. Then sperm suspension was rapidly drawn in and out by a 1ml pipette for 10 min to separate 60–80 % of sperm heads from their tails. To acquire oocyte-activating capacity decreased sperm, the sperm heads were incubated at 37℃ for 4 h. The other half of the top 1 ml sperm suspension was directly injected into oocytes for the control.

### Measurement of calcium oscillation pattern

Firstly, MII mouse oocytes were loaded in KSOM with 10µM fura-2 acetoxymethyl ester (fura-2 AM, Invitrogen) for 20 min at 37 ℃. The dye was first dissolved in DMSO containing Pluronic F-127 (Molecular Probes no. P3000) (final concentration, 0.08 % v/v and 0.016 %, respectively) and then diluted in KSOM-HEPES medium. After washing three times, the loaded oocytes were incubated in KSOM medium at 37 °C under 5 % CO_2_. Then the oocytes were used for ICSI or ICSI-AOA in different groups under the conditions described. And the oocytes were allowed at least 20 min of recovery in KSOM. Next, the oocytes were placed in drops of KSOM under mineral oil on an inverted microscope equipped with an atmospheric chamber and a filter switch providing excitation alternating between 340 and 380 nm for fura-2, and the emission wavelength is 510 nm. On average, 10-15 oocytes in each group were measured simultaneously. A scan was performed every 5 s over a time period of 1 h. The intracellular calcium levels were recorded in terms of the ratio of 340 and 380 nm fluorescence. Four different categories were distinguished according to the frequency of calcium oscillatory pattern as follows [16, 17]with appropriate changes: (0) no Ca^2+^ spikes, (+) 1–3spikes, (++) 4–8 spikes and (+++) > 8 spikes within 1 h following ICSI.

### Mouse ICSI and artificial oocyte activation (AOA)

The microinjection was performed on an inverted microscope (Olympus) equipped with a warm plate and a micromanipulation system (MM-89; Narishige, Iapan) as previously described [18]. The oocytes were treated with ionomycin (Sigma, St. Louis, MO, USA) in different concentrations for 10 min at 37 ℃ in 5 % CO2, after 30 min of ICSI. Our study contained 4 groups, as follows: (A, ICSI group) normal sperm injection as control, (B, ICSI-AOA group) normal sperm injection with AOA treatment, (C, dICSI group) oocyte activation-deficient sperm injection, (D, dICSI-AOA group) oocyte activation-deficient sperm injection with AOA treatment. To determine the optimal concentration for each treatment, the dICSI-derived oocytes were treated with concentrations of 0 (solvent control), 1µM, 2.5µM ionomycin for 10 min. The suitable AOA condition was determined by calcium oscillation pattern analysis compared with group A. Then the oocytes were thoroughly washed and left in KSOM for further culture or for the next calcium oscillation pattern analysis.

### Embryo transfer

Female ICR mice aged 8-12 weeks old were used as recipient mothers. The night before embryo transfer, they were mated with vasectomized ICR male mice to obtain pseudopregnant foster mothers. According to the presence of a vaginal plug, surrogate ICR mice were identified, and the day of the vaginal plug was designated as 0.5 d.p.c. (days post coitum). Each time, 14-18 embryos were transferred at the 2-cell stage into the oviduct of each recipient using a glass pipette. The days of fetal sampling were 6.5 d.p.c., 7.5 d.p.c., and 8 d.p.c., respectively. After birth, pups were weighed weekly until 8 weeks of age. Pups were weaned after the third week, and females and males were put separately. At 8 weeks of age, couples were formed by putting male and female offspring with fertile controls to assess their fertility.

### Collection of Samples and Transcriptome High-Throughput Sequencing

The blastocyst stage embryos were collected for sequencing. Five blastocysts were pooled for each treatment group (ICSI, ICSI-AOA and dICSI-AOA) in the same time from started injection and three independent replicates for each treatment procedure were collected. ICSI, ICSI-AOA and dICSI-AOA were named group A, B and D, respectively in the following sequencing. Total RNA was isolated from blastocyst of samples with TRIzol reagent (Life Technologies, Carlsbad, CA, USA) and GlycoBlue™ Coprecipitant (Ambion, AM9515) for facilitating good RNA recovery while increasing the size and visibility of the pellet. The RNA concentration of each sample was measured using the NanoDrop ND-1000 instrument (Thermo Fisher Scientific, Waltham, MA, USA). All RNA samples met standards of quality control according to the qualified ratio of OD260/OD280 ranged from 1.8 to 2.1. Transcriptome high-throughput sequencing was provided by CloudSeq Biotech Inc. (Shanghai, China). RNA preprocessing for constructing the sequencing library was implemented with the SMARTer Stranded Total RNA-Seq Kit (Takara). Libraries were controlled for quality and quantified using the BioAnalyzer 2100 system (Agilent Technologies, Santa Clara, CA, USA).

### Bioinformatics analysis

Paired-end reads were acquired from the Illumina HiSeq 4000 sequencer. After 3′ adaptor-trimming and removing low quality reads by cut adapt software (v1.9.3), the high quality trimmed reads were used to analyse mRNAs. The high quality reads were aligned to the mouse reference genome (UCSC mm10) with hisat2 software. Then, cuff diff software (part of cufflinks) was used to get the FPKM as the expression profiles of mRNA guided by the Ensembl gtf gene annotation file, and fold change and p-value were calculated according to FPKM, differentially expressed mRNA were identified. Alternative splicing was performed by SplAdder software. The statistical significance differentially expressed mRNAs (fold changes > 1.3 and p < 0.05) were identified. The data had been deposited in the National Center for Biotechnology Information (NCBI) Gene Expression Omnibus (GEO). The GEO accession number is GSE155948.

### RNA extraction and Q-PCR

Each group of blastocyst pool was separately collected and performed according to the Cell Amp Whole Transcriptome Amplification Kit (TaKaRa, Japan). cDNA was amplified through 20 cycles of PCR. Subsequently, 10-fold diluted cDNA was used as the template for Q-PCR, which was performed based on the SYBR Green I Real-Time PCR Kit (TaKaRa). The primers were presented in Supplementary Table S1. Each gene was tested in triplicate.

### DNA bisulfite treatment

Bisulfite mutagenesis was performed as previously described [15], with one blastocyst in each pool. Briefly, the DNA was wrapped in agarose gels to form little beads. The beads were treated with sodium bisulfite based on an EZ DNA Methylation Kit (Zymo Research, Orange, CA). The beads were immediately used for PCR or stored at −20 °C. Methylation-specific PCR (MS-PCR) primers were used to match the allele-specific DNA strands (Supplementary Table S1). Following nested PCR, cloning and sequencing were performed as previously described [15]. Briefly, the first round of PCR was conducted using one bead containing bisulfite-treated DNA with the outer primers and Taq HS premix (TaKaRa). For the second round of PCR, MS-PCR primers and 2 µl of the first PCR amplified products were used. To identify the specific amplification in PCR, 5 µl of each second round PCR product was separated by agarose gel electrophoresis. Next, the remaining PCR amplified products were cloned into a T vector (pMDTM19-T Vector Cloning Kit, TaKaRa) and individual clones were sequenced. The bisulfite sequencing PCR was also carried out according to the above protocols.

### Statistical analysis

All analyses were performed using SPSS, version 21.0 software (SPSS Inc., Chicago, IL, USA). Quantitative data are expressed as mean ± standard deviation (SD). Comparisons between groups were performed with t test or Chi-squared test. Principle component analysis was performed using ADE4 package of R according to the standardized expression profile data. Values of *P* < 0.05 were considered to be significantly different.

## Results

### Calcium oscillation pattern analysis

**Fig. 1:**
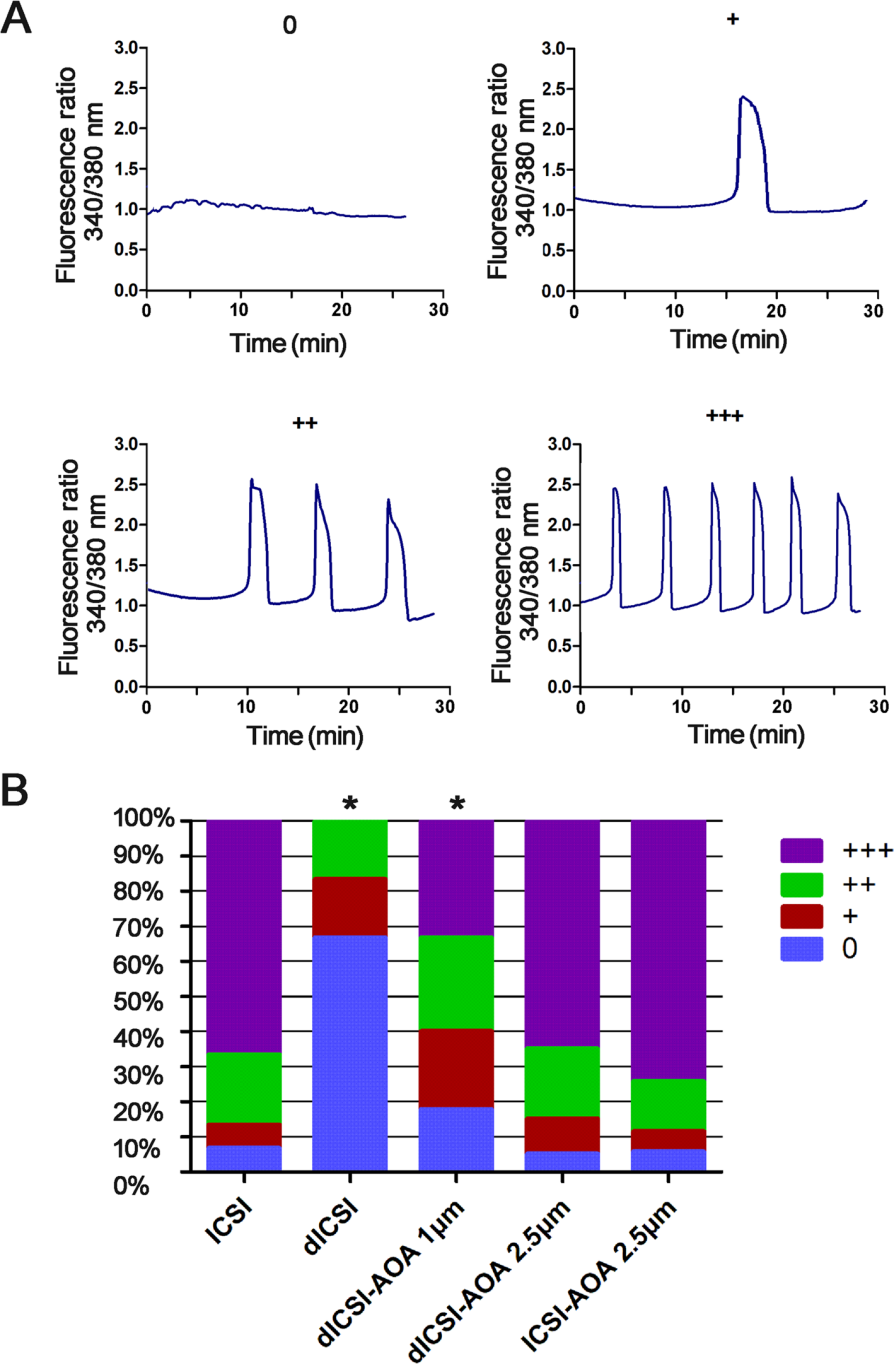
Calcium oscillation pattern analysis. **A** Representative calcium oscillation patterns for the calcium frequency scoring following normal mouse spermatozoa. The frequency score per oocyte is given based on the number of calcium rises seen during the 1 h measuring period: ‘+++’, > 8 spikes; ‘++’, 4–8 spikes; ‘+’, 1-3 spikes; ‘0’, no calcium spikes seen. **B** Frequency scoring of the calcium oscillations following normal mouse spermatozoa or oocyte activation-deficient mouse spermatozoa ICSI with different concentration of ionomycin. * indicates distribution of frequency pattern significant differences compared to ICSI group, *P* < 0.05

Firstly a standard calcium oscillatory profile was evaluated in the mouse oocytes with injection of normal sperm. The frequency of calcium oscillations was scored per oocytes within 1 h following ICSI (Table S2). Representative traces were shown in Fig. 1 A. We defined ≥ 1 Ca^2+^ spike/recording period triggered as ‘oscillatory activity’. Calcium oscillation analysis following ICSI with oocyte activation-deficient sperm (dICSI) demonstrated that 33.33 % of the injected mouse oocytes showed oscillatory activity, whereas 93.33 % of the injected oocytes using normal sperm showed a highly calcium rises (*P* < 0.05, Table S2). The average number of calcium oscillatory per responding oocytes injected with oocyte activation-deficient sperm was significantly lower (3.56 rises, range 1-8) than that of injected oocytes using normal sperm (9.52 rises, range 1-16) (*P* < 0.05, Table S1). Amplitude of calcium rises had no significant difference. No calcium rises were observed following the non-injected oocytes (n=10) or sham injection oocytes (n=10). We next explored the influence of AOA on the calcium oscillation pattern in mouse oocytes injected with oocyte activation-deficient sperm. 1µM and 2.5µM ionomycin were used for oocyte activation in order to choose the suitable AOA condition by calcium oscillation pattern analysis compared with control ICSI group (Table S3 & Fig. 1B). Frequency analysis in dICSI-AOA with different ionomycin concentrations demonstrated that the distribution of frequency patterns in 2.5µM ionomycin was comparable to that of ICSI group. Therefore we considered that at least 2.5 µM ionomycin was needed to support the oocyte activation-deficient sperm to generate normal Ca^2+^ response activity. So 2.5µM ionomycin was used for the following experiment.

### Effects of artificial oocyte activation treatment on the embryo development in vitro

**Fig. 2:**
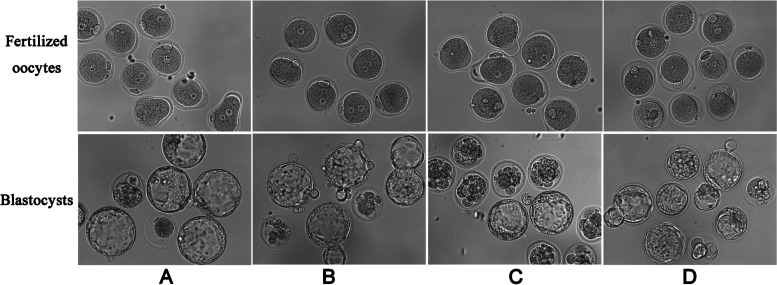
The representative images of early embryo development in four experimental groups: **A** ICSI group; **B** ICSI-AOA group; **C** dICSI group; **D** dICSI-AOA group

To determine the effect of AOA on the fertilization and early embryonic development of ICSI-derived embryos. AOA was performed following ICSI injected with normal sperm (B, ICSI-AOA group) or oocyte activation-deficient sperm (D, dICSI-AOA group). The normal sperm injection as control (A, ICSI group). The results showed in Table 1 indicated that the rate of fertilization decreased notably in oocyte activation-deficient sperm ICSI group (C, dICSI group) compared to ICSI group (30.8 % vs. 84.4 %, *P* < 0.05), which was rescued after AOA treatment. Furthermore, there was no statistically significant difference in the rate of fertilization between ICSI-AOA group and ICSI control group (83.1 % vs. 84.4 %, *P* > 0.05). And 2.5 µM ionomycin did not induce parthenogenetic activation of mouse oocytes. In addition, AOA treatment in group D has the higher blastocyst formation rate than that of dICSI-derived embryos (36.8 % vs. 10 %, *P* < 0.05). Moreover, there were no significant differences in the rate of blastocyst formation among group A, group B and group D (41.5 %, 40.7 % and 36.8 %, respectively, *P* > 0.05). The representative images of early embryo development were shown in Fig. 2. Thus, the results revealed that AOA treatment could improve the fertilization and blastocyst formation of oocyte activation-deficient sperm ICSI-derived embryos in vitro. Furthermore, moderate AOA treatment did not affect fertilization and embryo development in normal ICSI group.

**Table 1:** Effects of AOA treatment on the development of ICSI-derived embryos in vitro

Group	No. of surviving oocytes	No. of fertilization oocytes (%)	No. of blastocyst stage oocytes(%)
A, ICSI group	77	65(84.4)^a^	27(41.5) ^a^
B, ICSI-AOA group	71	59(83.1) ^a^	24(40.7) ^a^
C, dICSI group	65	20(30.8) ^b^	2(10) ^b^
D, dICSI-AOA group	72	57(79.2) ^a^	21(36.8) ^a^
No injection + AOA	18	0	0

a,b With the same column are significantly different (*P* < 0.05)

### Profiles of Differentially Expressed mRNAs

**Fig. 3:**
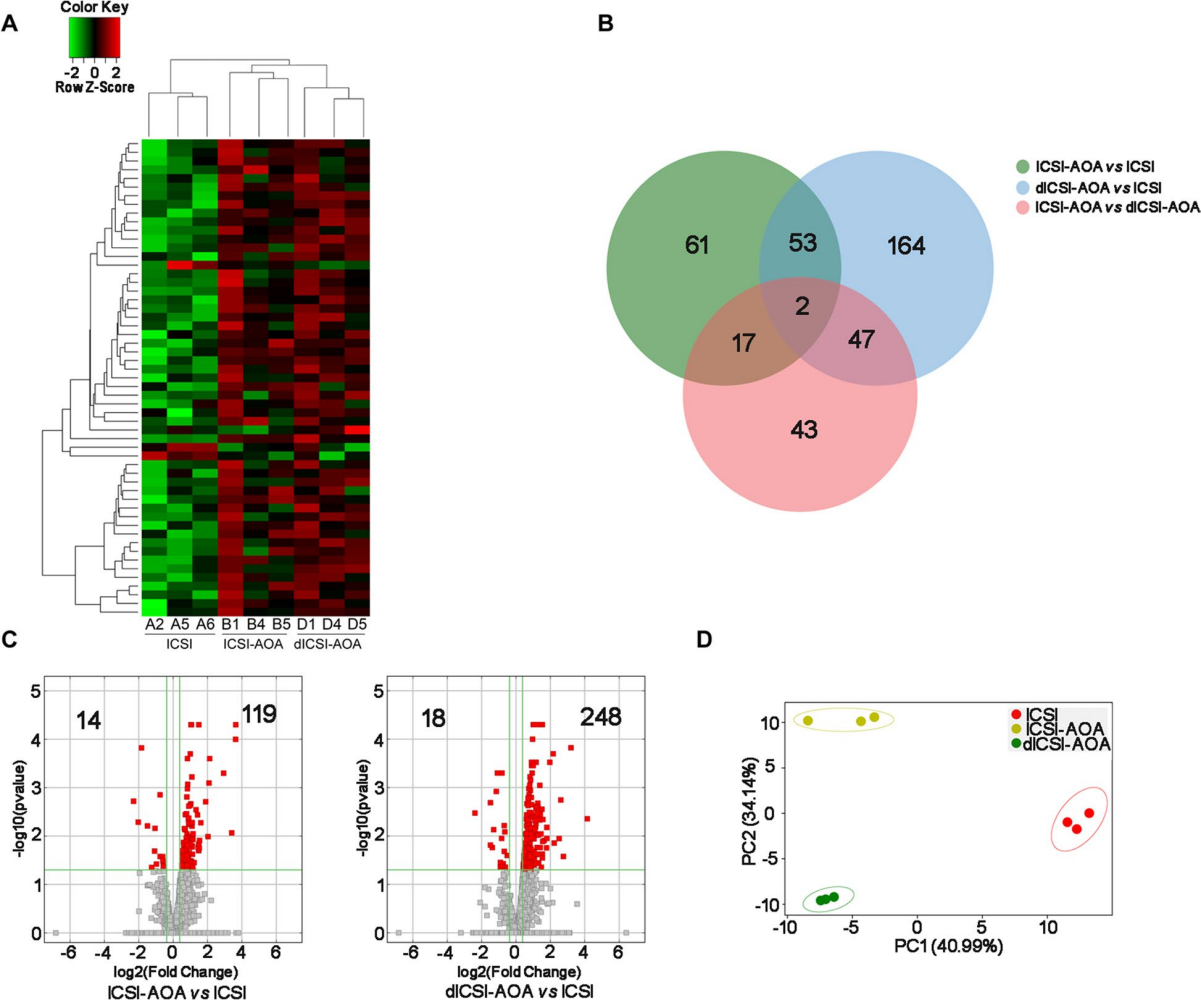
The mRNA expression analysis in blastocysts obtained in ICSI group, ICSI-AOA group and dICSI-AOA group. **A** Heatmap of global gene expression. Letters and numbers denote ID of a specific embryo. **B** Venn diagram with differentially expressed genes (DEGs) for pair-wise comparisons (ICSI-AOA vs. ICSI, dICSI-AOA vs. ICSI, ICSI-AOA vs. dICSI-AOA). **C** Volcano plot defined up-regulated/down-regulated mRNAs for pair-wise comparisons (ICSI-AOA vs. ICSI, dICSI-AOA vs. ICSI). **D** Principal Component Analysis (PCA) of the RNA-Seq samples: ICSI (red), ICSI-AOA (yellow) and dICSI-AOA (green)

The blastocyst stage embryos were collected for the sequencing. The sequencing analysis contained 3 groups, as follows: (A, ICSI group) normal sperm injection as control, (B, ICSI-AOA group) normal sperm injection with 2.5 µM ionomycin treatment, (D, dICSI-AOA group) oocyte activation-deficient sperm injection with 2.5 µM ionomycin treatment. After high-throughput sequencing, the heat map showed the hierarchical clustering of the distinguishable mRNA expression profiling among three groups (Fig. 3 A). Hierarchical clustering analysis using differentially expressed gene set make the samples into two major clustering branches. Three replicate samples in ICSI group self-cluster into one branch and the samples of ICSI-AOA and dICSI-AOA group cluster into another branch. Moreover, all the replicates of ICSI-AOA and dICSI-AOA group self-cluster into two major sub-branches. In the process of differential analysis of mRNAs, the results showed that there were 133 differentially expressed genes (DEGs) between the ICSI-AOA group and ICSI group, and 266 DEGs between the dICSI-AOA group and ICSI group (Fig. 3B). The DEGs for pair-wise comparisons was listed in table S4. There was a higher number of up-regulated (119/133-89.5 % in ICSI-AOA group and 248/266-93.2 %in dICSI-AOA group) than down-regulated (14/133-10.5 % and 18/266-6.8 %, respectively) in significantly different genes. Each dot stood for an mRNA in the volcano plot (Fig. 3 C). The red dots on the left side stood for the down-regulated mRNAs and those on the right side stood for the up-regulated mRNAs with significant differences, while the grey dots stood for the mRNAs which expressed differentially without significance. Principal Component Analysis (PCA) distributed samples into a two dimensional space according to the variance in gene expressions, and samples with similar trends in gene expression profiles will cluster together in the PCA plot. The result showed that 9 samples clearly segregate into three clusters, corresponding to ICSI, ICSI-AOA and dICSI-AOA group (Fig. 3D). PCA analysis demonstrated that the embryos from each group clustered together, despite expected individual variability.

### Expression of imprinted genes in embryos with artificial oocyte activation treatment

**Fig. 4:**
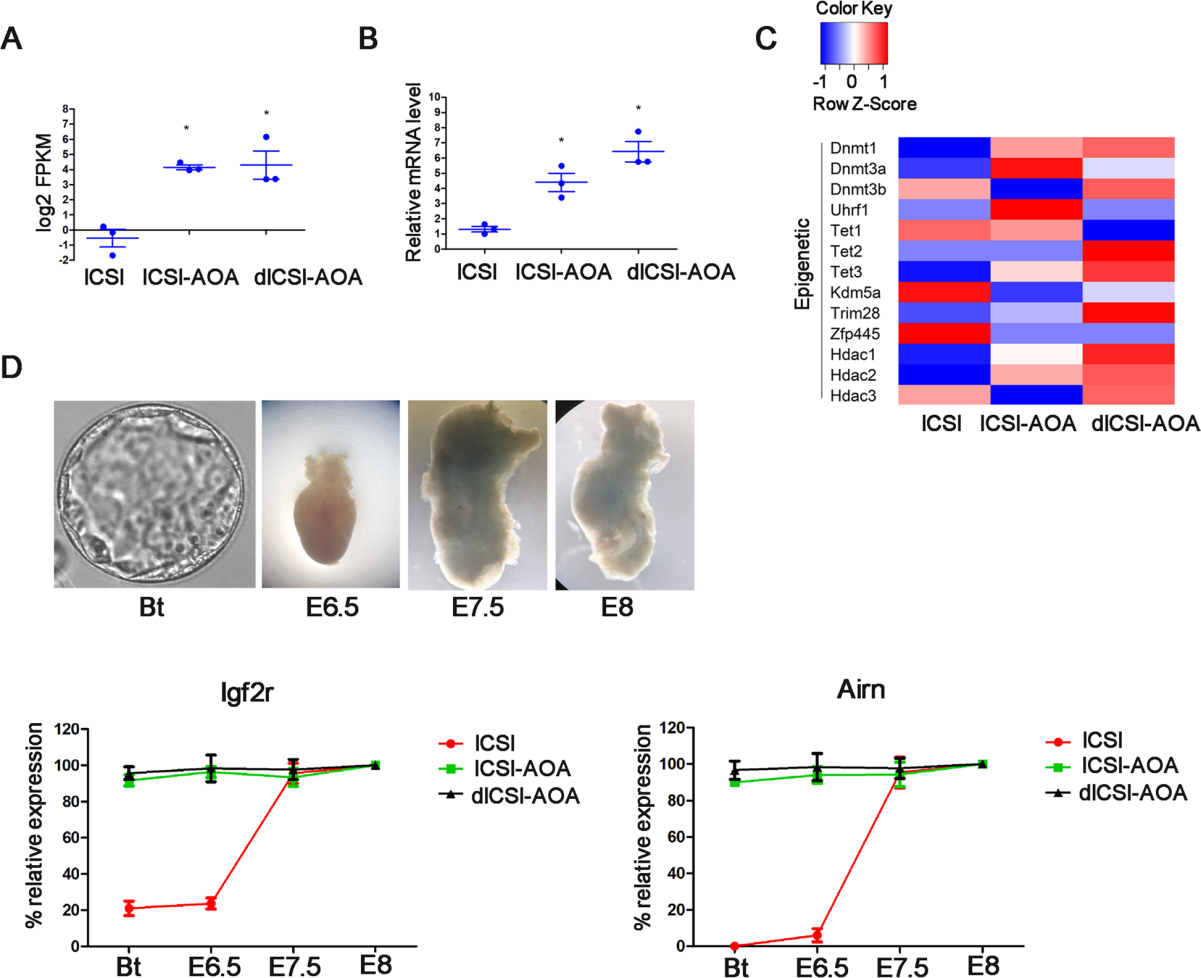
The imprinted gene expression in embryos. **A** Transcript abundance of *Igf2r* imprinted genes in blastocysts obtained after ICSI, ICSI-AOA or dICSI-AOA. Each spot represents one experiment. **B** Relative expression level of *Igf2r* imprinted genes was validated by quantitative PCR. Each spot represents one experiment. * *P* < 0.05 was indicated significantly different. **C** Heat map of expression of key genes related to epigenetic reprogramming. **D** QPCR analysis of gene expression at Bt, E6.5, E7.5, and E8. Left panel showed *Igf2r* expression during embryo development stages. Right panel showed *Airn* expression during embryo development stages. The value at E8 was set to 100. The mean of three biological replicates is given with SD. Bt, blastocyst

To investigate the epigenetic changes of AOA method in ART technology, we evaluated the effect of AOA treatment on the imprinted genes expression. In the process of differential analysis of imprinted genes, there were two differentially expressed imprinted genes between ICSI-AOA group and ICSI group, and one differentially expressed imprinted gene between dICSI-AOA group and ICSI group. *Igf2r* was both up-regulated in ICSI-AOA group (FC=22.786) and dICSI-AOA group (FC=38.849) compared to that of ICSI group. The expressions of *Igf2r* in three groups were validated by quantitative PCR, which was consistent with the sequencing results (Fig. 4 A&B). Moreover, in order to evaluate the effect of AOA manipulation on the expression of genes associated with epigenetic reprogramming, we analyzed the expression of key genes related to methylation, demethylation and remethylation process. The result was presented in the heat map in Fig. 4 C. Meanwhile, we compared these genes in DEGs. Despite of some genes with a wide range in transcript abundance, there were no significant differences in these gene expressions for pair-wise comparisons.

### Methylation states of ***Igf2r*** in blastocysts derived from artificial oocyte activation treatment

**Fig. 5:**
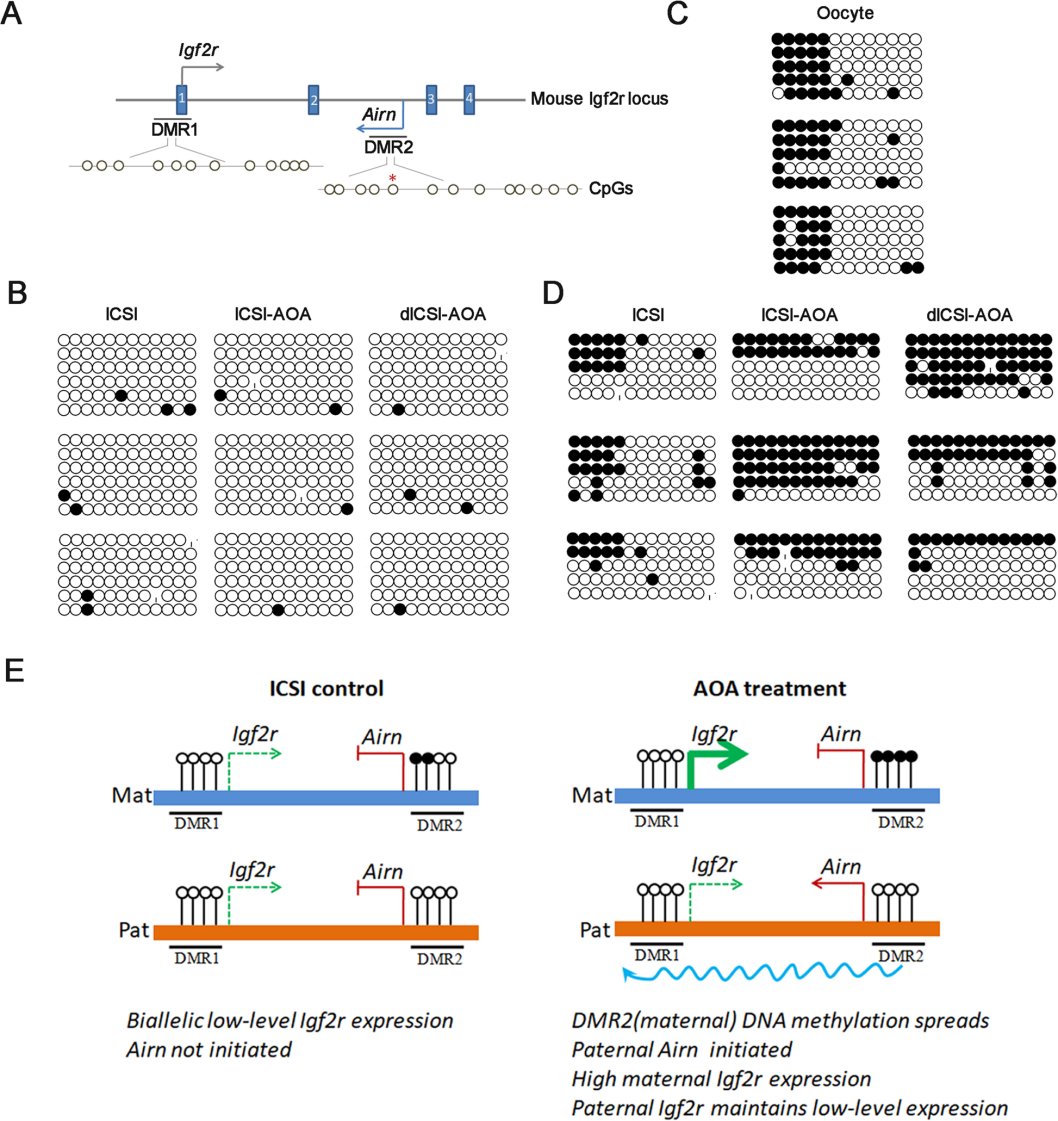
DNA methylation state at DMRs. **A** Schematic diagram of mouse *Igf2r/Airn* locus: solid boxes are represent *Igf2r* exons, and location of DMR1 and DMR2. 12 and 13 CpG dinucleotides were analyzed. Open circles indicates CpG islands. The methylation boundary in oocytes was marked by red asterisk. **B** DNA methylation profiles of *Igf2r* DMR1 in mouse blastocysts. The analyzed region contains 12 CpG sites. Per ‘mini-array’ indicates one blastocyst and six clones were sequenced for each array of the group. **C** DNA methylation profile of *Airn* DMR2 in mouse oocytes. The analyzed region contains 13 CpG sites and five clones were sequenced for each array. **D** DNA methylation profile of *Airn* DMR2 in mouse blastocysts. The analyzed region contains 13 CpG sites and five clones were sequenced for each array of the group. Open circles indicate unmethylated CpG sites and filled circles indicate methylated CpG sites. Missing circles are represent CpG sites whose methylation status could not be determinate. Horizontal lines represent one individual clone. Lollipop diagrams were generated using BIQ Analyser software. **E** Summary schematic depiction on dynamic regulation of *Igf2r*/*Airn* in preimplantation embryo. In ICSI control blastocysts, *Igf2r* is expressed biallelically (dashed arrow) and *Airn* is silent. The DMR1 is unmethylated on both parental alleles. The DMR2 is methylated on the maternal allele and unmethylated on the paternal allele. In AOA treatment ICSI blastocysts, maternal methylation at DMR2 spreads, followed by initiation of paternal *Airn* transcription. *Igf2r* is up regulated expression on the maternal allele (thick green arrow), but the up-regulation is blocked on the paternal allele by *Airn* transcriptional interference in *cis*

The *Igf2r* locus has two known DMRs (Fig. 5 A). The methylation profiles of the imprinted genes *Igf2r* promoter (DMR1) were analyzed in blastocysts obtained from three experimental groups. The number of 12 CpG sites were examined in the *Igf2r* promoter. The results were presented in a ‘lollipop’ format (Fig. 5B). The sequencing results were derived from unmethylated-specific primers. When we used methylated-specific primers, no PCR products were obtained. We concluded that DMR1of *Igf2r* showed unmethylated status on both parental alleles in these three groups (Fig. 5B). The results suggest that AOA treatment does not affect DMR1 DNA mehtylation. From another point of view, it can be explained that DNA mehtylation in DMR1 does not regulate the expression of *Igf*2*r* in preimplantation embryo.

### Dynamic regulation of ***Igf2r/Airn*** in the pre-implantation embryo

Figure S1 depicts the previous known of *Igf2r/Airn* imprinted expression model. In preimplantation embryos or undifferentiated ES cells, *Igf2r* is expressed biallelically and *Igf2r* promoter is unmethylated, *Airn* is silent but the maternal *Airn* promoter carries a DNA methylation imprint. In late post-implantation or late differentiated ES cells, *Igf2r* is up-regulated on the maternal allele, but the up-regulation is blocked by *Airn* transcriptional interference in *cis* on the paternal allele. The paternal *Igf2r* maintains low-level expression and gains DNA methylation in late post-implantation or late differentiation. Since *Airn* is considered to regulate the expression of *Igf2r*, we examined the relative mRNA expression levels of *Airn* in three groups at blastocyst stage. The relative expression results were shown in Fig. 4D. *Airn* was expressed in AOA-derived blastocyst. However, no *Airn* was detected in control ICSI-derived blastocyst. These results indicate that AOA treatment might initiate *Airn* transcription to regulate the *Igf2r* expression.

To further explore the methylated regulation of *Igf2r/Airn*, we next analyzed the methylation in DMR2. DMR2 methylation has been proved to be present in oocytes. Previous reports demonstrated DMR2 present the precise 3’ boundary of ICR mehtylation [CpG at Chr17:12,742,488-12,742,489] in oocytes. Next, the methylation profiles of DMR2 including 3’boundary was analyzed in oocytes. We examined 13 CpG sites in DMR2. The results showed that the precise ICR border was maintained in oocytes (Fig. 5C). Next, the methylation profiles of DMR2 including 3’ boundary was analyzed in blastocysts obtained from three experimental groups. In ICSI control blastocysts, the precise 3’ ICR border was detected in some clones. However, DNA methylation showed spreading in the 3’direction in AOA treatment blastocysts (Fig. 5D). These results indicated that methylation at DMR2 may increase or spread in cells of preimplantation embryo with AOA treatment. In order to better explore the methylation of specific allele, the methylation-specific PCR (MS-PCR) primers in two flanks of 3’ boundary were designed for DMR2 methylation analysis in three experiment groups (Table S1). We examined 10 CpG sites and 7 CpG sites in DMR2, respectively (Figure S2). For the left side of 3’ boundary, the maternal allele was methylated allele, the paternal allele was unmethylated allele. For the right side of 3’boundary, the DNA strand containing the methylated allele was not detected using methylated-specific primers in ICSI group. In other words, the precise 3’ border was maintained on the maternal allele in ICSI control blastocysts. However, DNA methylation had spread in the 3’ direction in AOA treatment blastocysts. These results indicated that maternal allele-specific methylation at DMR2 increases or spreads in pre-implantation embryo with AOA treatment.

In our model, Fig. 5E showed a summary schematic depiction about regulation of *Igf2r*/*Airn* in blastocyst embryos obtained from three groups. In ICSI control blastocysts, *Igf2r* is expressed biallelically and DMR1 is unmethylated on both parental alleles. *Airn* is silent and the maternal DMR2 carries a DNA methylation imprint region in which the precise ICR border mehtylation was maintained. In AOA treatment ICSI blastocysts, maternal DMR2 methylation spreads, followed by the initiation of paternal *Airn* transcription. *Igf2r* is up regulated expression on the maternal allele, but the up-regulation is blocked by *Airn* transcriptional interference in *cis* on the paternal allele. The paternal *Igf2r* maintains low-level expression. It is showed that the extends of DMR2 methylation is coincident with initiation of *Airn* expression in AOA treatment blastocysts, demonstrating that AOA treatment stimulates maternal allele-specific mehtylation spreads at DMR2, followed by initiation of paternal *Airn* transcription, resulting up regulated expression of *Igf2r*.

### Expression of ***Igf2r/Airn*** in the post-implantation embryo

To further explore the expression of *Igf2r/Airn* in the post-implantation embryo with AOA treatment, we transferred ICSI, ICSI-AOA and dICSI-AOA embryos into surrogate mothers. Next, we used QPCR to quantify the expression of *Igf2r* and *Airn* at E6.5, E7.5, and E8. Figure 4D showed that *Igf2r* and *Airn* expression increased during embryo development stages: a sharp increase was observed between E6.5 and E7.5 for both genes in ICSI control group. Relative to blastocysts, *Igf2r* increased on average ~5-fold and *Airn* increased ~100-fold in ICSI control group. However, the expression of *Igf2r* and *Airn* did not change in AOA treatment group during embryo development stages. Relative to ICSI control blastocysts, the gene expression in AOA treatment embryo at blastocyst and E6.5 was the same as that of ICSI control embryo at E7.5. These results further demonstrated that AOA treatment promoted *Airn* expression in advance, followed by regulation of *Igf2r* expression.

### Post-implantation development following artificial oocyte activation treatment

The post-implantation development of embryos obtained from the three groups were presented in table S5. The newborn rates per pregnant recipient were similar. The birth weight of the newborn mice derived from AOA treatment embryos was significantly lower when compared with the newborn mice from ICSI derived embryos. The pups born following AOA treatment did not show any other abnormalities during early development. Moreover, the weight in all study groups was measured weekly up to 8 weeks of age (Figure S3). No significant differences were found in male or female pups among all study groups. In addition, all the weaned mice grew to adulthood. At 8 weeks of age, the offspring were mated to fertile WT mice and the mice born following AOA treatment (both female and male) mated successfully with healthy offspring born.

## Discussion

Infertile couples with oocyte activation deficiency may experience ICSI failure. ICSI plus AOA has been proven to be an effective method to mitigate fertilization failure problems. However, little is known whether AOA can alter gene expression in human pre- and post-implantation embryonic development. Due to the current ethical constraints, as well as the scarcity of clinical samples, and thus clinical experiments are too difficult to carry out. The use of animal model would help to study the safety of oocyte activation, avoiding the effect of parental circumstances and genetic backgrounds. In our previous study, the oocyte activation-deficient mouse spermatozoa was identified [14]. With the release of the sperm-associated oocyte-activating factors from disrupted membranes, spermatozoa with different oocyte activation-deficient were established. This spermatozoa model could be used to study fertilization mechanisms, and to identify novel oocyte-activation strategies.

In our study, 66.67 % oocytes showed no calcium rises after injection of activation-deficient sperm. Ionomycin is a commonly used assisted activator in some clinics [19], we used different concentrations of ionomycin to explore its optimal intensity. Frequency (F) reflected the total number of Ca2+ spikes per recording period. The calcium oscillations begin a few minutes after gamete fusion [20], occur at various frequencies and cease at the time of pronucleus (PN) formation, i.e. 4-6 h later [21, 22]. The frequency analysis demonstrated that the frequency of calcium oscillation will increase synchronously with the increase of AOA intensity and the distribution of frequency patterns in 2.5µM ionomycin was comparable to that in ICSI group. This means that activated deficiency of sperm could be rescued by appropriate AOA treatment. At present, a wide variety of patients with various causes of infertility may be employed by AOA treatment, which was not because of the oocyte activation deficiency [23–25]. Meanwhile, for normal sperm, we also used 2.5µM ionomycin after ICSI to explore the effect of excessive activation on embryos. The analyses of calcium oscillations showed that the extra activation (2.5µM ionomycin) did not impact the distribution of frequency patterns (Fig. 1B). To better verify the accuracy of the analysis, the duration of a single calcium oscillation was analyzed in all study groups. Duration of the every Ca2+ transient was measured as the time from when the first sharp change in positive slope occurs to when it returned to the same point (Figure S4A). The results of the two methods were consistent (Figure S4B).

It is known that fertilizing sperm induce oscillations in Ca^2+^ levels to initiate the activation of oocytes, and intracellular calcium signaling also plays an essential role in the sequential embryonic development [26]. In addition, a close correlation between cell division and calcium availability has been reported [27, 28], that alteration in Ca^2+^ signaling may be the underlying reason for defects in cell growth and cleavage. Furthermore Wong et al. showed that the application of ionophore could overcome the negative effects caused by Ca^2+^ deficiency, e.g. cleavage furrow regression [29]. Our previous study also proved that the incubation of sperm heads at 37 °C led to defects in fertilization and embryo development potential, which could be rescued with AOA [14]. Moreover, Ebner et al. reported that AOA with Ca^2+^ ionophore could improve blastocyst formation rate and clinical outcomes in infertile patients with previous embryo developmental problems [24]. In Fig. 2; Table 1, the blastocyst formation rate of group C was significantly lower than that of group D. The results revealed that AOA treatment could improve the blastocyst formation rate of activation-deficient sperm ICSI-derived embryos in vitro, which were consistent with the previous studies.

Previous studies indicated that the embryo is sensitive to its very early environment in ART and may have long-lasting consequences [30, 31]. With the rapid development of genome-wide transcriptome analysis, an increasing number of studies have been focusing on the different gene expression following ART manipulation. Now RNA and DNA sequencing have become more useful technologies that could help to study the expression changes observed in AOA-derived embryos. For the first time, our results provide a profile of mRNA expression in early mouse embryos with AOA treatment. A total of 54 mRNAs, among which 51 were up-regulated and 3 were down-regulated, were significantly altered in both two AOA treatment groups (ICSI-AOA group & dICSI-AOA group) compared to ICSI group. These genes are marked in yellow in the supplement table S4. As described in our result, appropriate AOA treatment did not cause a large number of gene expression changes compared with ICSI group. The noteworthy pathways during oocyte activation were inositol phosphate metabolism and phosphatidyl inositol signaling system pathway. The KEEG analyses of these two pathways were showed in supplement Figs. 5 and 6. Oocyte activation is a Ca^2+^-dependent process. And Ca^2+^ ionophores used for AOA treatment give high permeability to cell membranes allowing Ca^2+^ ions to penetrate through. This Ca^2+^ influx from the intracellular stores, particularly the ER, induce an increase of free intracytoplasmic Ca^2+^ in oocytes [32]. Moreover, ionomycin might exert the substantial cellular stress by altering membrane permeability, which may differ from the physiological Ca^2+^ oscillatory response [33, 34]. It is possible that these different events following ICSI-AOA trigger a different wave of gene activation, especially phosphatidyl inositol signaling pathway. In addition, a higher number of up-regulated than down-regulated genes presented in AOA treatment group, suggesting that this artificial activation step stimulate some events initiated by sperm penetration.

It is known that imprinted genes play an important role in regulating the growth and development of embryo and placenta in utero, and also in the early post-natal phase. Some non-physiological embryo culture environments or manipulation in vitro may influence the epigenetic modification of imprinted genes during early embryo-genesis [35]. Some epigenetic changes in pre-implantation embryos may further affect gene expression during fetal development stage [36].Therefore, we focused the study to explore the effect of AOA on expression of imprinted genes. In the mouse, there are about 150 known imprinted genes, many of which occur in imprinted gene clusters that are regulated together. The maternally expressed *Igf2r*, *Slc22a2*, and *Slc22a3* genes and the paternally expressed lncRNA *Airn* were presented in one of the gene cluster on mouse chromosome 17 [37]. Imprinting of *Igf2r* gene is controlled by DMR2 that contains the promoter of the long non-coding (lnc) RNA *Airn*, whose transcript overlaps the paternal *Igf2r* promoter in an antisense orientation [38]. *Igf*2*r* is initially biallelically expressed from four-cell stage to the blastocyst and does not show maternal-specific expression until implantation [39, 40]. The expression of *Igf2r* in somatic tissues is related to the DMRs on the parental chromosomes, which DMR1 includes the *Igf2r* promoter, and DMR2 is located in intron 2 of *Igf2r*, encompassing the *Airn* promoter. DMR2 acquires its maternal methylation during oogenesis, whereas DMR1 acquires paternal methylation after post-implantation[41]. In ICSI control blastocysts, the precise ICR border in maternal allele was maintained. However, DNA methylation had spread in the 3’direction in AOA treatment blastocysts. The results indicated that DMR2 maternal allele methylation increases or spreads in pre-implantation embryos with AOA treatment, which were consistent with that occurring during gastrulation (E6.5-E7.5). This study proposed that AOA treatment may stimulates maternal allele-specific mehtylation spreads in advance at DMR2, followed by initiation of paternal *Airn* transcription, resulting up regulated expression of *Igf2r*.

The previous studies proved that reduction of *Igf2r* gene expression has functional consequences for increased birth weight [42] and the biallelic *Igf2r* expression (+/R2Δ), compared with wild-type mice that have imprinted maternal-specific expression, has the decreased weight of mice [43]. Post-implantation development results indicated that the lower birth weight observed in neonates of AOA groups compared with that of ICSI group, which may be the result of IGF2R overexpression in advance. Nevertheless, no significant differences were found in post-natal growth of the pups in present study.

## Conclusions

The aim of this study was to determine the effect of AOA treatment on the gene expression in the developing mouse embryo. In this study, we provide a profile of the changes in the global patterns of gene expression in ICSI-AOA treatment versus ICSI generated mouse blastocysts. Another key observation in this study is that AOA treatment affects imprinted gene *Igf2r* expression, which is regulated by the imprinted *Airn* lncRNA. It is hoped that the study could provide valuable insights of AOA technology in assisted reproduction biology. And further studies are needed to evaluate the long-term effect of AOA on the offsprings.

## Supplementary Information

**Additional file 1: Figure S1.** Dynamic regulation of Igf2r/Airn in the preimplantation embryo. What was previously known? In preimplantation embryos or undifferentiated ES cells, Igf2r is expressed biallelically (dashed arrow) and Igf2r promoter is unmethylated on both parental alleles (white oval). Airn is silent, and promoter is methylated on the maternal allele (black hexagon) and unmethylated on the paternal allele (white hexagon). In late post-implantation or late differentiated ES cells, Igf2r is up-regulated on the maternal allele (thick green arrow), but its up-regulation is blocked on the paternal allele by Airn transcriptional interference in cis. The paternal Igf2r promoter gains DNA methylation in late post-implantation or late differentiation (black oval). Airn (wavy line) is transcribed from the unmethylated paternal allele. References: 1(Szabo and Mann,1995), 2(Lerchner and Barlow, 1997), 3(Wang et al., 1994), 4(Stoger et al., 1993), 5(Braidotti et al., 2004), 6(Mikkelsen et al.,2007), 7(Latos, P.A.,2009), 8(Sleutels et al., 2002), 9(Latos, P.A., et al.2012), 10(Marcho, C., et al. 2015).

**Additional file 2: Figure S2.** Airn DMR2 MS-methylation in two flanks of 3’ boundary. (A) DMR2 MS-methylation state in the left side of 3’ boundary in mouse blastocysts. The analyzed region contains 10 CpG sites and five clones were sequenced for each array of the group. (B) DMR2 MS-methylation state in the right side of 3’ boundary in mouse blastocysts. The analyzed region contains 7 CpG sites and five clones were sequenced for each array of the group.

**Additional file 3.** Postnatal growth of the pups. Postnatal growth of male (A) and female (B) pups during the first 8 weeks after birth obtained following ICSI (green), ICSI-AOA (red), or dICSI-AOA (blue). Data are Mean±SD. X-axis: time in weeks; Y-axis: body weight in g.

**Additional file 4: Figure S4.** Duration of the every Ca2+ transient. (A) Schematic showing how duration times were measured. (B) Graph comparing the mean duration times of a single Ca2+ transients in all groups. Asterisks indicate significant differences compared to ICSI control group.

**Additional file 5: Figure S5.** Iositol phosphate metabolism by The Kyoto Encyclopedia of Genes and Genomes (KEGG) analysis.

**Additional file 6: Figure S6.** Posphatidylinositol signaling system by The Kyoto Encyclopedia of Genes and Genomes (KEGG) analysis.

**Additional file 7.**

## Data Availability

All data generated through this study are included in this article.
