# Supplementary figures and images for: DNA methylation and gene expression changes in mouse pre- and post-implantation embryos generated by intracytoplasmic sperm injection with artificial oocyte activation
Source: Reprod Biol Endocrinol. 2021 Nov 4;19:163. doi: 10.1186/s12958-021-00845-7 (PMC8567642; doi:10.1186/s12958-021-00845-7)

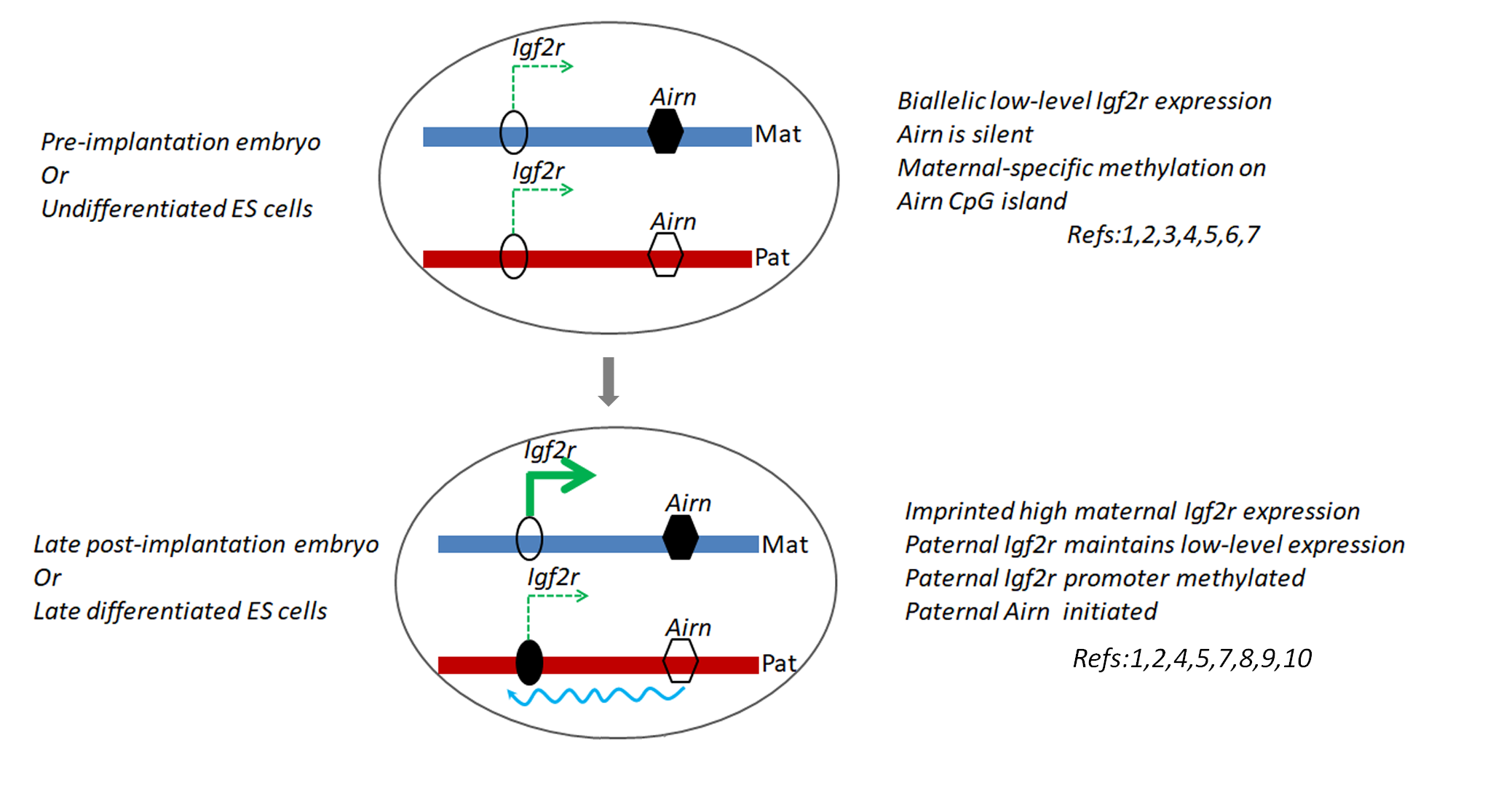

Supplement: Supplementary file 1 — Additional file 1: Figure S1. Dynamic regulation of Igf2r/Airn in the preimplantation embryo. What was previously known? In preimplantation embryos or undifferentiated ES cells, Igf2r is expressed biallelically (dashed arrow) and Igf2r promoter is unmethylated on both parental alleles (white oval). Airn is silent, and promoter is methylated on the maternal allele (black hexagon) and unmethylated on the paternal allele (white hexagon). In late post-implantation or late differentiated ES cells, Igf2r is up-regulated on the maternal allele (thick green arrow), but its up-regulation is blocked on the paternal allele by Airn transcriptional interference in cis. The paternal Igf2r promoter gains DNA methylation in late post-implantation or late differentiation (black oval). Airn (wavy line) is transcribed from the unmethylated paternal allele. References: 1(Szabo and Mann,1995), 2(Lerchner and Barlow, 1997), 3(Wang et al., 1994), 4(Stoger et al., 1993), 5(Braidotti et al., 2004), 6(Mikkelsen et al.,2007), 7(Latos, P.A.,2009), 8(Sleutels et al., 2002), 9(Latos, P.A., et al.2012), 10(Marcho, C., et al. 2015). [file 12958_2021_845_MOESM1_ESM.tif]

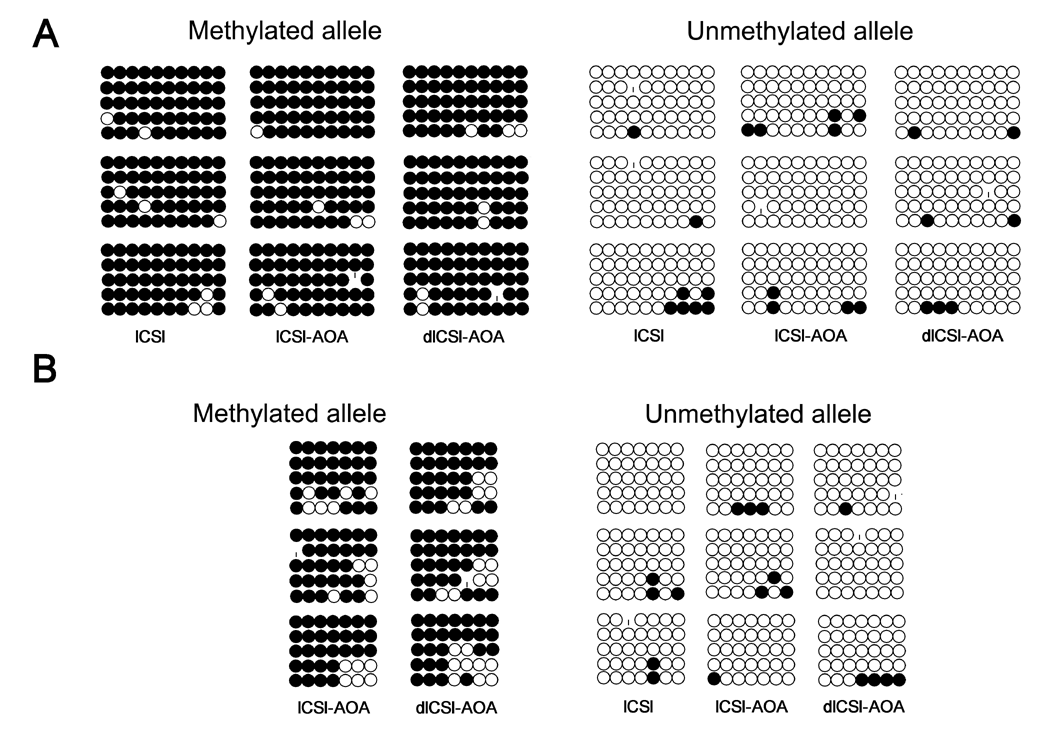

Supplement: Supplementary file 2 — Additional file 2: Figure S2. Airn DMR2 MS-methylation in two flanks of 3’ boundary. (A) DMR2 MS-methylation state in the left side of 3’ boundary in mouse blastocysts. The analyzed region contains 10 CpG sites and five clones were sequenced for each array of the group. (B) DMR2 MS-methylation state in the right side of 3’ boundary in mouse blastocysts. The analyzed region contains 7 CpG sites and five clones were sequenced for each array of the group. [file 12958_2021_845_MOESM2_ESM.tif]

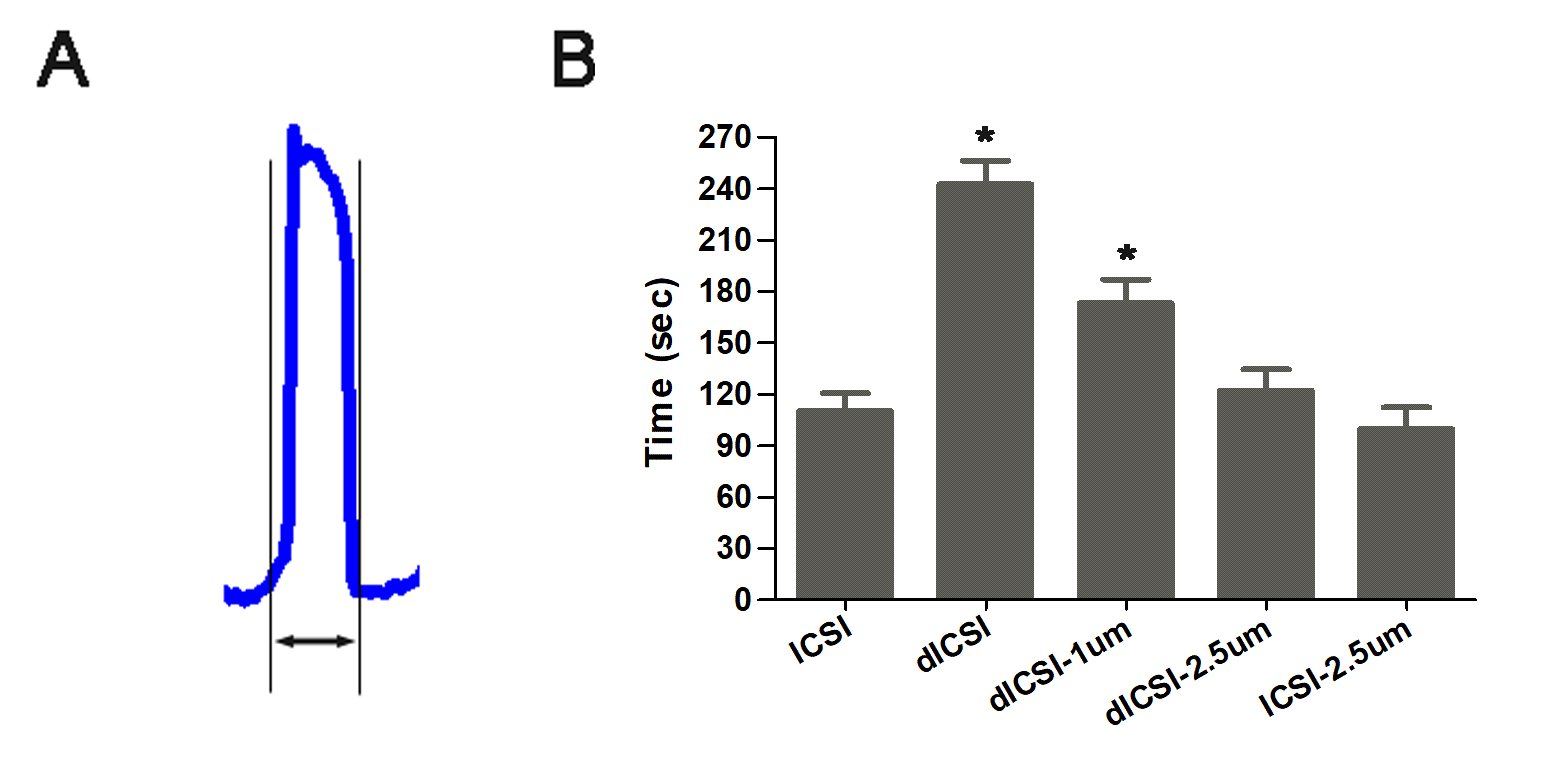

Supplement: Supplementary file 4 — Additional file 4: Figure S4. Duration of the every Ca2+ transient. (A) Schematic showing how duration times were measured. (B) Graph comparing the mean duration times of a single Ca2+ transients in all groups. Asterisks indicate significant differences compared to ICSI control group. [file 12958_2021_845_MOESM4_ESM.tif]

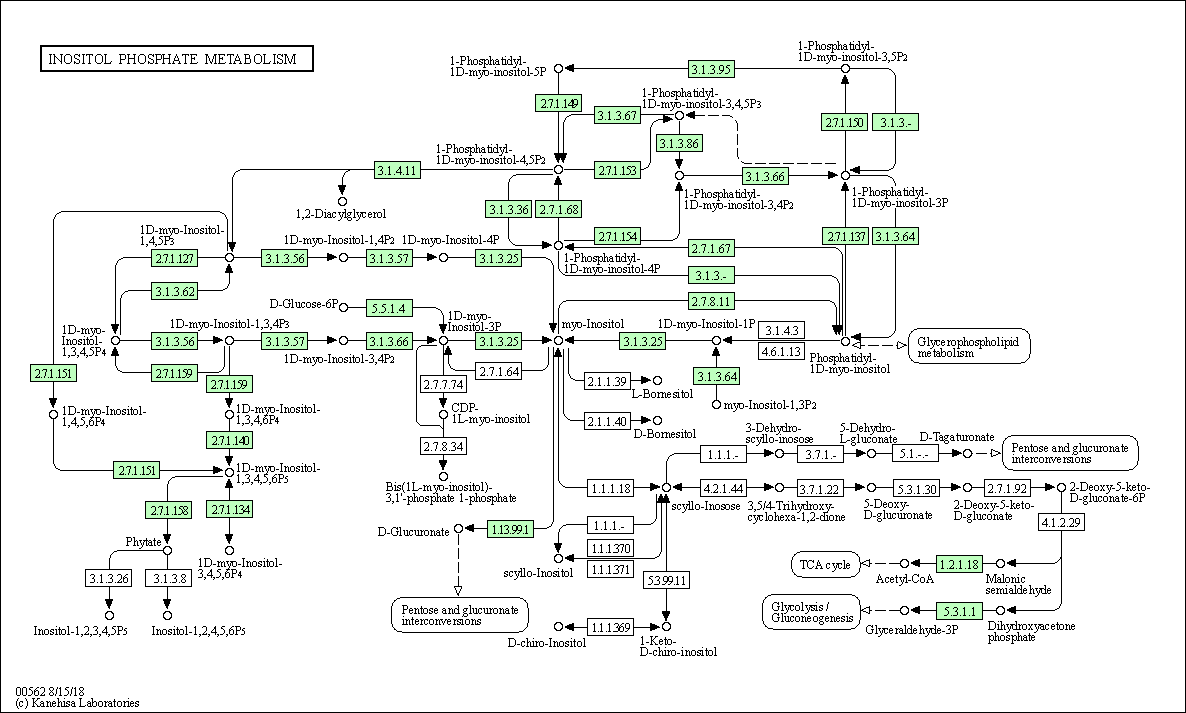

Supplement: Supplementary file 5 — Additional file 5: Figure S5. Iositol phosphate metabolism by The Kyoto Encyclopedia of Genes and Genomes (KEGG) analysis. [file 12958_2021_845_MOESM5_ESM.png]

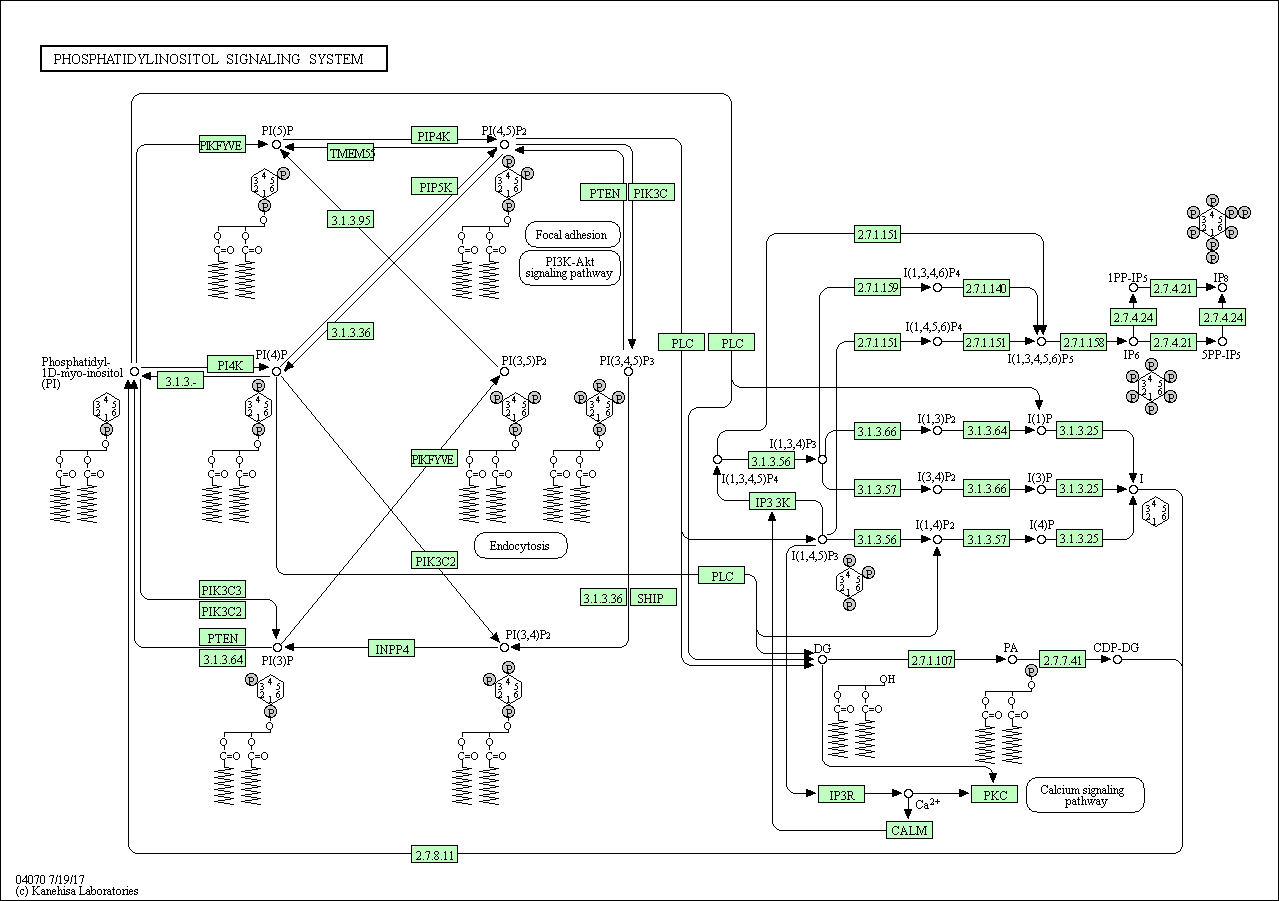

Supplement: Supplementary file 6 — Additional file 6: Figure S6. Posphatidylinositol signaling system by The Kyoto Encyclopedia of Genes and Genomes (KEGG) analysis. [file 12958_2021_845_MOESM6_ESM.png]
