# Supplementary material for: DNA methylation and gene expression changes in mouse pre- and post-implantation embryos generated by intracytoplasmic sperm injection with artificial oocyte activation
Source: Reprod Biol Endocrinol. 2021 Nov 4;19:163. doi: 10.1186/s12958-021-00845-7 (PMC8567642; doi:10.1186/s12958-021-00845-7)
Supplement: Supplementary file 7 — Additional file 7. [file 12958_2021_845_MOESM7_ESM.zip › supl table.docx]

Table S1 Sequence of primers used in the real-time PCR and bisulphate methylation-specific PCR analyses.

| Gene | Accession | Primer type | Primer sequence (5′-3′) |
| --- | --- | --- | --- |
| Mouse *Igf2r* | 16004 | *Igf2r*_F | TTCGACCTATAAGAAGCCTT |
|  |  | *Igf2r*_R | GGGTACTTTGCTTTTGGGTA |
|  |  | Outside F | TTGGGTTTTTTTATTTAATTTTATTT |
|  |  | Outside R | AACCTAACAACCCCAAAATTACTC |
|  |  | Methylated-specific F | TTATTTCGGTTATCGTATTGGTTTC |
|  |  | Methylated-specific R | ACAACCCCAAAATTACTCACG |
|  |  | Unmethylated-specific F | AATTTTATTTTGGTTATTGTATTGGTTTT |
|  |  | Unmethylated-specific R | TAACAACCCCAAAATTACTCACAC |
| Mouse *Airn* | 104103 | *Airn*_F | GGGTGGAGCCTTATGATGAA |
|  |  | *Airn*_R | TGAAGCCTGGGTTTCATTTC |
|  |  | O-F | TTAAGGGTGAAAAGTTGTATAAGGAG |
|  |  | O-R | ACAAAACCCTCTAAATCCTCCTATC |
|  |  | I-F | GGGTTGTGATTTTGGTTATGTTAAG |
|  |  | I-R | CACCTTCATTTACATAACCAATAAAAA |
|  |  | Outside F1 | GAGGTGAGGGTTTTATTGATT |
|  |  | Outside R1 | AAACCCTACCCTTATACAACTTC |
|  |  | Methylated-specific F1 | TAGTATAATTTTAATTGTGTTGCGA |
|  |  | Methylated-specific R1 | AAATTACTCCGAACCCTCGAA |
|  |  | Unmethylated-specific F1 | AGGATTTTAGTATAATTTTAATTGTGTTGT |
|  |  | Unmethylated-specific R1 | AAATTACTCCAAACCCTCAAA |
|  |  | Outside F2 | GGGTTGTGATTTTGGTTATGTTAAG |
|  |  | Outside R2 | AACCAATAAAAAACCCTAATCTTAC |
|  |  | Methylated-specific F2 | TGTAAATTGTATAAGGGGAGGATTC |
|  |  | Methylated-specific R2 | ACCCTCATACATAACCAAAATAACG |
|  |  | Unmethylated-specific F2 | TAAATTGTATAAGGGGAGGATTTGA |
|  |  | Unmethylated-specific R2 | CCCTCATACATAACCAAAATAACAC |

F, forward; R, reverse.

Table S2 Calcium oscillation pattern analysis

|  | n | Oocytes with calcium rises, n (%)* | Calcium rises per responding oocytes, n ± SD | Amplitude of calcium rises, mean ± SD |
| --- | --- | --- | --- | --- |
| ICSI | 45 | 42(93.33%)^a^ | 9.52±3.15^a^ | 2.40±0.05 |
| dICSI | 48 | 16(33.33%)^b^ | 3.56±2.22^b^ | 2.38±0.05 |
| Sham ICSI | 10 | 0 | 0±0 | 0±0 |
| Non-injected oocytes | 10 | 0 | 0±0 | 0±0 |

a-b Values with different superscripts within same column are significantly different (*P* < 0.05)

* defined with ≥ 1 Ca^2+^ spike/recording period

Table S3 The number of oocytes in different Ca^2+^ oscillations patterns following normal mouse spermatozoa or oocyte activation-deficient mouse spermatozoa ICSI with different concentration of ionomycin.

|  | No. of oocytes | | | |
| --- | --- | --- | --- | --- |
|  | 0 | + | ++ | +++ |
| ICSI (n=45) | 3 | 3 | 9 | 30 |
| dICSI (n=48) | 32 | 8 | 8 | 0 |
| dICSI-AOA 1μM (n=45) | 8 | 10 | 12 | 15 |
| dICSI-AOA 2.5μM (n=40) | 2 | 4 | 8 | 26 |
| ICSI-AOA 2.5μM (n=35) | 2 | 2 | 5 | 26 |

Frequency pattern of Ca^2+^ oscillations: ‘+++’ >8; ‘++’ 4–8; ‘+’ 1–3; ‘0’, absence of Ca^2+^ oscillations.

Table S5 Post-implantation development.

| Group | Transferred  embryos, n | Recipients, n | Newborn rate per pregnant recipient^a^, (%) | Birth weight,  Mean±SD |
| --- | --- | --- | --- | --- |
| ICSI | 65 | 4 | 21 (32.3%) | 1.44±0.05 |
| ICSI-AOA | 75 | 5 | 25 (33.3%) | 1.34±0.05* |
| dICSI-AOA | 72 | 5 | 22 (30.6%) | 1.33±0.06* |

^a^ pups/transferred embryos

* With the same column are significantly different (P＜0.05)
